# Effects of Double-Taped Kinesio Taping on Pain and Functional Performance due to Muscle Fatigue in Young Males: A Randomized Controlled Trial

**DOI:** 10.3390/ijerph17072364

**Published:** 2020-03-31

**Authors:** Haneul Lee, Hyoungwon Lim

**Affiliations:** 1 Department of Physical Therapy, Gachon University, Inchon 21936, Korea; leehaneul84@gachon.ac.kr; 2 Department of Physical Therapy, Dankook University, Cheonan 31116, Korea

**Keywords:** functional performance, kinesio taping, muscle fatigue, pain, tension

## Abstract

Kinesio taping (KT) is widely applied for pain control and rehabilitation in clinical settings. Tape tension is a key factor in the taping method. However, limited evidence exists regarding the reinforced tension effects of KT on functional performance and pain in healthy individuals. This study aimed to investigate the immediate effects of double-taped Kinesio taping (DTKT) on functional performance and pain caused by muscle fatigue after exercise. A total of 44 healthy male students (mean age, 23.3 ± 2.2 years) were randomly assigned to the following three groups: DTKT, normal-tape Kinesio taping (NTKT), and placebo. The single-hopping (SH) distance, vertical jump height (VJH), and power (VJP) were assessed at baseline. The muscle fatigue protocol was then applied to induce muscle soreness. Outcome measures including subjective pain, SH distance, VJH and VJP were evaluated immediately after the muscle fatigue protocol, and KT was then applied; the measures were then again evaluated immediately and 24 h after KT application. No significant interactions between pain and functional performance were observed (*p* > 0.05), and there were no significant differences in SH, VJH, and VJP among the groups (*p* > 0.05). Notably, the DTKT had an immediate effect on the alleviation of pain caused by muscle fatigue. The present findings indicate that DTKT is not superior to NTKT or placebo in terms of pain relief and enhancing functional performance after tape application in healthy male students.

## 1. Introduction

Kinesio taping (KT) is one of the most common adhesive therapeutic taping technique and has been shown to be clinically effective for improving the range of motion [1,2,3] and muscle activities [4,5], shortening of an earlier occurrence of muscle peak torque generation [6,7], and enhancing functional performance [8,9,10].

In various sports and exercise activities, vertical jumping is a well-known movement [11,12]. Quick extension of the lower limb joints, including the hip, knee, and ankle, occurs before beginning the push-off movement. The vertical jump height (VJH) is an essential factor for sports performance that is often considered an indicator of an individual’s ability to participate in exercise or sports activities. [13]. Vertical jumping can be used as exercise training to gain strength and increase power of the lower extremities. Vertical jumping-associated movements comprise actions at multiple joints, including the hip, knee, and ankle, with active contraction of the major muscles such as hamstrings, quadriceps, triceps surae, and muscles of the lower back [14].

In athletes, especially those who perform jumping, vertical jumping movements exhibit relatively normalized patterns in terms of complex movements of multiple joints requiring explosive muscle contraction, which results in maximal muscle performance of the lower joints [15]. Therefore, jumping can be performed to improve muscle strength and endurance of the lower extremities [16]. Several researchers claimed that functional performance can be enhanced by applying KT [2,3]. KT reduces inflammation and promotes joint movement by improving blood flow and lymph circulation [17]. It also helps relieve pain by decreasing pressure on subcutaneous nociceptors and facilitates joint and muscle function by improving sensory feedback and muscle activation for prevention of injuries, rehabilitation, and enhancement of functional performance [2,3,18]. Huang et al. observed significantly better improvements in high-jump performance during the single-leg hop (SH) test and in vertical ground reaction force and enhanced muscle activity of the medial gastrocnemius during vertical jump test with KT than with placebo tape in healthy inactive men [13]. 

However, the effects of KT on functional performance, especially on muscular strength and endurance, remain controversial because of limited clinical and scientific evidence [2,3,18]; therefore, no clear scientific agreement has yet been reached regarding the effect of KT on athletes’ performance in terms of muscular strength and endurance. Nevertheless, many Olympic athletes use KT to improve their performance, and the trend is becoming increasingly popular [19,20].

Some studies have demonstrated that KT application does not immediately enhance functional performance of healthy individuals without pain due to muscle fatigue, regardless of subject deception and changes in tape tension [14,21,22,23]. This might be because these participants are pain-free and their functional performance is good. However, in individuals with pain caused by muscle fatigue, the functional performance is affected by KT, and KT might help reduce pain. 

Neuromuscular activation and proprioception play a key role in maintaining joint function and stability [24]. A previous study suggested that the tension provided by the tape can improve proprioceptive feedback and facilitate correct posture and movement, even after the removal of the tape, which can improve muscle activities for functional performance [25]. Joint proprioception can be enhanced with external supports such as elastic band or taping [24,26]. 

However, Long et al. showed that KT on the foot and ankle may amplify sensory input, which enhances proprioception of poor ankle performers, but produces an input overload that impairs proprioception in those who were originally good performers [27]. 

Thus, tape tension is a critical element of the KT method [14]; however, previous studies applied only the stretched tape to increase tension but not the double-taped technique to reinforce tension [28].

Therefore, the present study aimed to compare the effects of double-taped KT (DTKT), normal-tape KT (NTKT), and placebo taping on functional performances such as SH distance, VJH and vertical jump power (VJP), and on subjective pain of the lower extremities in individuals with muscle fatigue. We hypothesized that KT with double tension would reduce pain, which would improve functional performance. 

## 2. Materials and Methods 

### 2.1. Ethical Approval

This study was approved by the institutional review board of Gachon University, registered on the clinicaltrials.gov website (KCT 0003522) and conducted in accordance with the Consolidated Standards of Reporting Trial recommendations. All participants signed a statement of informed consent prior to participation in the study.

### 2.2. Participants

A total of 50 male students were recruited in this study; among these, 2 declined to participate in the study and 4 did not meet the inclusion criteria; finally, 44 participants (mean age, 23.3 ± 2.2 years) were included. All participants were physically active and healthy, with a body mass index (BMI) of <25 kg/m^2^ (mean BMI, 23.3 ± 2.7 kg/m^2^) and practice time of >150 min of moderate or intense physical activity per week (mean physical activity time, 262.6 ± 48.5 min per week). Participants who had not received pharmacological therapy or undergone surgery of the lower extremities were eligible for the study, whereas those who had orthopedic, musculoskeletal, or neurological limitations of the lower extremities, and those who presented with signs of allergy or any inconvenience to KT were excluded.

The sample size was calculated using G power 3.0.1. software (Heinrich Heine University Dusseldorf, Dusseldorf, Germany). A sample size of 39 participants (13 participants per group and a 0% attrition rate) was estimated using a moderate effect size of f = 0.25, significance level of 0.05, and power of 0.85. Thirty-nine participants were required to show statistical significance when a clinically significant interaction was observed between the time points and groups. An additional five participants were recruited to provide for unanticipated attrition.

### 2.3. Procedure

**Figure 1:**
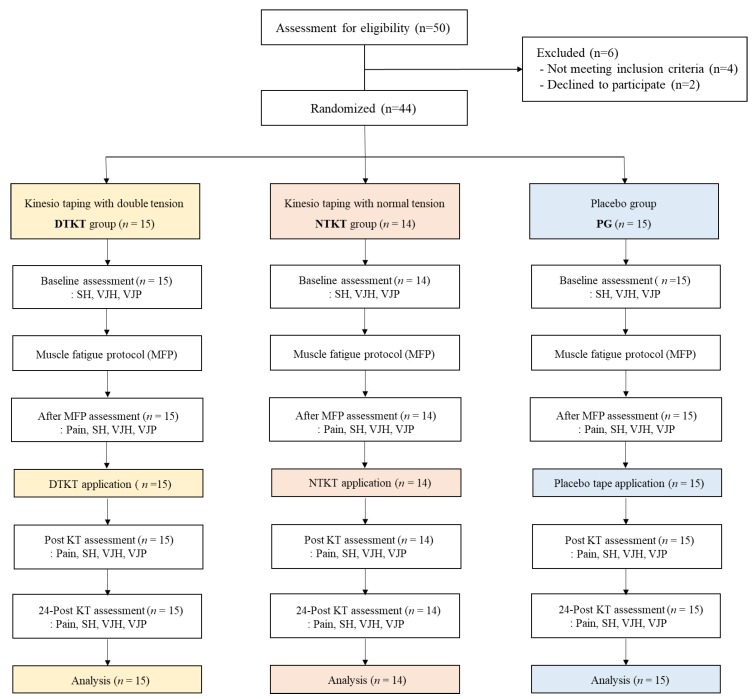
Flowchart diagram of the study. Abbreviations: SH, single hopping; VJH, vertical jump height; VJP, vertical jump power.

Simple randomization was independently conducted by one of the researchers, and concealed allocation was performed using a computer-generated randomized table of numbers created before data collection. Participants were randomly divided into the following three groups: DTKT (*n* = 15), NTKT (*n* = 14), and placebo (*n* = 15) groups (PG; Figure 1). This study was double-blinded, and different researchers performed randomization (researcher 1), assessment (researcher 2), intervention (researcher 3), and data analysis (researcher 4) to minimize potential bias.

Once participants arrived at the research laboratory, they were asked to sit comfortably for 10 min to stabilize their body condition at ambient room temperature. General characteristics (height, weight, and BMI) were measured before the beginning of the test. All participants were asked to perform static stretching of their lower extremities (calf muscles and quadriceps) for 5 min to warm up while they watched an exercise video that demonstrated countermovement jumps and SH for familiarization. Subsequently, the baseline SH distance and maximum VJH and VJP were measured. To induce muscle soreness, participants underwent the muscle fatigue protocol (MFP) under the supervision of researchers. The SH distance, VJH, and VJP were again measured after the MFP, and KT was applied. Outcome measurements were taken immediately (post-KT) and 24 h (24-post KT) after KT application. Subjective pain was also measured, except at baseline. Participants were asked not to perform more than moderate physical activities during 24 h between measurements, and if they did so, they were asked to report them. 

### 2.4. Muscle Fatigue Protocol 

The MFP basically included 20 bilateral squats (90° knee flexion) for 5 sets and 20 eccentric contractions of the ipsilateral calf muscles for 5 sets; however, if the participants reached the criteria of muscle fatigue, they would complete the MFP. For eccentric contractions of the ipsilateral calf muscles, participants were asked to stand on a step with the dominant leg and lower the heel over 3 s until they could not lower the heel any further. This MFP was modified based on previous studies [29,30] and determined according to the pilot study findings. 

Prior to the MFP, participants were informed about the modified Borg’s scale of perceived exertion (scale: 0–10) [31], which was used between the protocol sets to quantify the perceived exertion [29,32]. Participants performed the MFP until they reached the perceived exertion scale of 8 out of 10, and they were then asked to continue the MFP until they were fully exhausted [32,33].

### 2.5. Intervention (Applying Kinesio Taping)

The ATEX Kinesio sports tape (Atex Medical Co., Seoul, Korea) was applied on clean and dry skin of participants. The length of the KT applied was calculated using anthropometric dimensions and was defined by a change in length of the tape between the origin and insertion points of the rectus femoris and gastrocnemius before and after stretching [18,28].

KT has muscle facilitation effects when it is applied from the direction of origin to insertion points with 75% of the maximum length tension [6,21,34]. However, in the present study, KT was applied from the direction of insertion to origin points with 75% of the maximum length tension to investigate its muscle inhibition effect [18]. 

**Figure 2:**
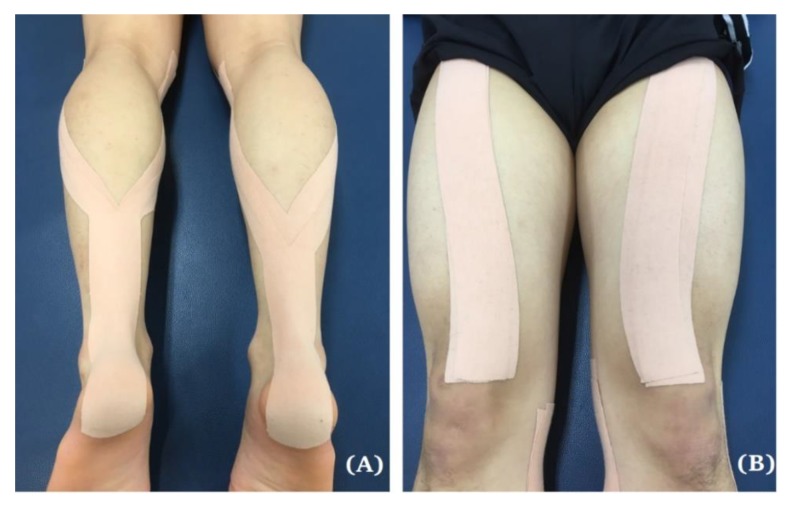
(**A**) Kinesio taping with double-taped application to the calf muscle; (**B**) Kinesio taping with double-taped application to the rectus femoris.

In the DTKT group, double-layer KT was applied to the participants while they were in a relaxed prone position, from the surface of the calcaneus bone on the sole of the foot to the end of origin of the medial and lateral gastrocnemius muscles below the knee joint using the Y-shaped taping technique (Figure 2A). The I-shaped taping technique was applied from the tibial tuberosity to the anterior superior iliac spine for the rectus femoris (Figure 2B). Participants in the NTKT group underwent the same procedures as those in the DTKT group, except that the KT was applied only in a single layer. In addition, participants in the PG underwent the same procedures as those in the NTKT group, except that they were asked to stay in a neutral position without tension to the CaduMedi non-woven adhesive sham tape (T&C Healthcare Co. Ltd., Guangzhou, China). All participants were instructed not to remove the applied tapes for 24 h until reassessment. If the tape was removed in 24 h, participants were asked to contact the researchers; indeed, no one had removed the tape within this time period. 

### 2.6. Outcome Measures

Subjective pain was measured using the visual analog scale for pain [35,36]. Participants were asked to place a vertical mark across a 10-point horizontal line, with the 10-point level indicating the greatest pain [17]. Only 1 scale was marked on each piece of paper so that any bias from previous measures could be eliminated.

The SH and vertical jump tests have been used as reliable methods to assess functional performance [15,37,38]. All measurements were taken with the dominant leg. In the SH test, participants started with single-leg standing with their hands on their waists behind a line on the floor and the knees slightly flexed for a while; then, they were asked to jump forward as quickly as possible and land with the same leg [37]. In the vertical jump test, participants were asked to stand at hip’s width, with their hands on their waists, and then asked to jump up from the ground as fast and high as possible with a verbal cue; the body was to remain stretched while jumping [39]. The VJH and VJP were calculated in this test.

The mean value of three attempts was calculated. The SH distance and VJH, and VJP in the SH and vertical jump tests, respectively, were assessed using the OptoGait system (Microgate Srl, Bolzano, Italy). The OptoGait system is a floor-based photocell system for movement analysis and functional assessment consisting of two 1-m transmitting and receiving bars, including a 96-light emitting diode (LED) that communicates via an infrared frequency and a web camera. The OptoGait system was placed 1.5 m apart on a flat surface, and the system calculated the time when the participants touched the floor or stayed in the air and communicated this information by sending and receiving 1000 signals per second, generating accurate data. The OptoGait software (Microgate Srl, Bolzano, Italy) was then used to calculate the precise VJH (cm) and VJP (w/kg) with the acquired data using the following [40,41]:

(1) $$h=\frac{T_{v}{}^{2}\;\cdot \;g\;}{8}\;p=g^{2}\cdot T_{v}\cdot \frac{\left(T_{v\;}+{\;T}_{c}\right)}{4\;\cdot T_{c}}$$

h = height (cm), p = power (W/kg), g = gravity acceleration, T_v_ = flight time, and T_c_ = contract time.

### 2.7. Statistical Analysis

The SPSS 23.0 software for Windows 10 was used for data analysis (IBM, Armonk, NY, USA). All data are presented as mean and standard deviation. Normality of the continuous variables was examined using the Shapiro–Wilk test, and all outcome variables were normally distributed. One-way analysis of variance (ANOVA) was conducted to compare general characteristics and baseline SH distance, VJH, and VJP among the three groups. A 3 (group) × 3 (time) two-factor mixed ANOVA was conducted to determine whether any interaction existed between the groups and time points. As no interaction was observed between the groups and time points in all outcome variables, one-way repeated-measures ANOVA was conducted to determine any differences in outcome variables among the time points in each group. Post hoc comparisons using the least significant difference (LSD) test were performed when significant group main effects were detected. The level of significance was set at *α* = 0.05.

## 3. Result

All 44 participants completed the study (15 in the DTKT group, 14 in the NTKT group, and 15 in the PG). General characteristics of the participants and baseline functional performance, including SH, VJH, and VJP, were not significantly different among the three groups (Table 1).

No significant difference was found in subjective pain after the MFP among the groups (F_[2,43]_ = 1.26, *p* = 0.30). No significant interaction in subjective pain was observed between the three time points (after MFP, post-KT, and 24-post KT) and groups (F_[3.1,41]_ = 0.54, p = 0.67, *η*^2^ = 0.03). Only a significant change in the mean subjective pain was found in the DTKT group from immediately after the MFP to post-KT (3.5 ± 1.9 to 2.5 ± 1.6, *p* = 0.004; Table 2).

The post-MFP SH distance was not significantly different among the groups (F_[2,43]_ = 0.40, *p* = 0.68), and a significant decrease in the mean SH distance after the MFP compared to baseline was observed in all three groups (*p* <0.01). No significant interaction was observed between the three time points (after the MFP, post-KT, and 24-post KT) and groups (F_[13.14,41]_ = 0.32, *p* = 0.86, *η*^2^ = 0.02; Table 3). There was no significant difference in the mean VJH after the MFP among the groups (F_[2,43]_ = 1.52, *p* = 0.23). In addition, the mean VJH was lower after the MFP than at baseline in each group (*p* < 0.01). No significant interaction was observed between the time points and groups (F_[3.4,41]_ = 1.55, *p* = 0.20, *η*^2^ = 0.07; Table 3). The VJP results were similar to the SH distance and VJH results. No significant difference in the mean VJP after the MFP was found among the groups (F_[2,43]_ = 0.70, *p* = 0.50). A significant decrease in the mean VJP after the MFP was found in all three groups (*p* < 0.05). In addition, no significant interaction in the mean VJP was found between the time points and groups (F_[2.4,41]_ = 0.86, *p* = 0.45, *η*^2^ = 0.04; Table 3).

## 4. Discussion

Tape tension is a key factor of the KT method [14]. One study showed that tape tension can improve proprioceptive feedback and facilitate correct posture and movement, even after removal of the tape [25]. However, another study found that the tension might have reversal effects on functional performance [27].

To our knowledge, the present study is the first to investigate the immediate effects of the DTKT technique, which provides more tension, on functional performance including the SH distance, VJH, and VJP of the lower extremities with pain due to muscle fatigue.

Our results indicate that there were no significant interactions in pain, SH distance, VJH, and VJP between the groups and time points (after the MFP, post-KT, and 24-post KT). This finding is consistent with that of previous studies that reported that KT application did not immediately enhance functional performance in healthy individuals, regardless of subject deception and changes in tape tension [14,21,22]. On the other hand, Mendez-Rebolledo et al. found that KT increased VJH and ground reaction force only 72 h, but not 24 h, after KT application on the gluteus maximus, biceps femoris, longissimus, rectus femoris, and gastrocnemius muscles in male athletes [22].

One potential mechanism of KT is that motor neuron threshold reductions can be induced by skin irritation, resulting in easier recruitment of motor units and improved functional performance [42]. In addition, KT is effective in pain control management by inhibiting muscle activities [1,3,19].

Functional performance was more related to muscle strength in the Konishi’s study and they showed that the tactile input from KT could inhibit muscle weakness and decrease muscle activities owing to decreased Ia afferents; however, tactile stimulation from KT was not enough to increase muscle strength in individuals without any injuries [43]. Our results indicate that KT, even with double layers, does not increase muscle performance or suppress pain caused by muscle fatigue because it might not be still enough to stimulate the non-nociceptive fibers. Our results support those of a previous study that reported that the tactile input from KT was not strong enough to improve muscle power, which influences VJH [14].

It is difficult to conclude that the effect of KT application is simply due to the placebo effect because its effects on pain, disability, and functional performance have been controversial over a decade [1,3,4,13,18,19,21,23,25,44,45,46].

The present study has several limitations. First, although tension is a key factor of KT according to a previous study [14], the tension of KT could not be quantified. Second, participants in this study were healthy individuals; therefore, our findings may not be applicable in clinical practice of athletic training and sports medicine. Third, KT was applied only on the rectus femoris and calf muscles to investigate functional performance of the lower extremities; however, more muscles of the lower extremities, such as the gluteus maximus, biceps femoris, tibialis anterior, multifidus, and peroneus are involved in high-speed jumping. Thus, data obtained might not provide accurate evidence on the effect of KT on functional performance. Fourth, this study did not include a control group without any taping. Therefore, further studies should include a control group without any taping to confirm our findings.

Despite these limitations, this study has several strengths: it is the first study to investigate the immediate effects of the KT technique reinforced with tape tension on the functional performance of the lower limbs and pain due to muscle fatigue in healthy individuals, and our study may provide the basis for further research on KT with reinforced tension.

## 5. Conclusions

In conclusion, the DTKT technique has immediate effects on the alleviation of pain caused by muscle fatigue. However, this technique is not superior to NTKT or placebo in terms of pain relief and functional performance within 24 h after tape application in healthy male students.

## Figures and Tables

**Table 1:** General characteristics of participants (*N* = 44).

	DTKT (*n* = 15)	NTKT (*n* = 14)	PG (*n* = 15)	*p* Values
Age (years)	23.2 ± 2.0	23.2 ± 2.7	23.4 ± 2.1	0.98
Height (cm)	172.9 ± 4.3	171.6 ± 7.6	174.3 ± 4.5	0.45
Weight (kg)	69.6 ± 8.4	69.6 ± 12.7	69.5 ± 8.2	0.99
BMI (kg/m^2^)	23.2 ± 2.4	23.6 ± 3.2	22.9 ± 2.4	0.80
Baseline SH distance (cm)	173.1 ± 16.1	178.6 ± 13.3	180.5 ± 15.0	0.40
Baseline VJH (cm)	31.5 ± 4.7	30.3 ± 5.4	33.7 ± 6.1	0.30
Baseline VJP (w/kg)	15.4 ± 2.5	15.9 ± 3.6	15.7 ± 2.5	0.97

Abbreviations: SH, single hopping; VJH, vertical jump height; VJP, vertical jump power; DTKT, double-taped Kinesio taping; NTKT, normal-taped Kinesio taping; PG, placebo group; BMI, body mass index.

**Table 2:** Subjective pain changes after intervention in the three groups (*N* = 44).

		DTKT (*n* = 15)	NTKT (*n* = 14)	PG (*n* = 15)	F ^1^	P ^1^	*η* ^2 1^
Pain	After MFP	3.5 ± 1.9	3.5 ± 2.9	2.3 ± 1.8	0.54	0.67	0.03
	Post	2.5 ± 1.6 *	2.9 ± 1.8	2.1 ± 1.9			
	24-post	3.3 ± 2.4	3.0 ± 2.3	2.2 ± 2.2			

^1^ F, P, and η^2^: interaction between time and group. * significant decrease after MFP. Abbreviations: DTKT, double-taped Kinesio taping; NTKT, normal-taped Kinesio taping; PG, placebo group; MFP, muscle fatigue protocol.

**Table 3:** Functional performance changes after intervention in the three groups (*N* = 44).

		DTKT (*n* = 15)	NTKT (*n* = 14)	PG (*n* = 15)	F ^1^	P ^1^	*η* ^2^ ^1^
SH distance (cm)	Baseline	173.1 ± 16.1	178.6 ± 13.3	180.5 ± 15.0			
	After MFP	163.9 ± 20.0 *	165.0 ± 20.7 *	170.3 ± 22.3 *	0.32	0.86	0.02
	Post KT	168.2 ± 15.6	165.9 ± 21.4	170.8 ± 23.9			
	24-post KT	168.6 ± 14.1	166.3 ± 20.7	172.2 ± 23.1			
VJH (cm)	Baseline	31.5 ± 4.7	30.3 ± 5.4	33.7 ± 6.1			
	After MFP	29.1 ± 4.6 *	26.2 ± 5.5 *	29.8 ± 7.3 *	1.55	0.20	0.07
	Post KT	28.6 ± 3.7	26.9 ± 6.7	30.9 ± 6.8			
	24-post KT	29.8 ± 4.2	27.3 ± 6.3	29.4 ± 8.5			
VJP (w/kg)	Baseline	15.4 ± 2.5	15.9 ± 3.6	15.7 ± 2.5			
	After MFP	14.4 ± 1.0 *	13.8 ± 2.8 *	14.7 ± 2.3 *	0.86	0.45	0.04
	Post KT	14.3 ± 1.0	14.2 ± 3.7	14.8 ± 2.4			
	24-post KT	15.9 ± 1.2	14.9 ± 4.2	14.7 ± 3.5			

^1^ F, P, and *η*^2^: interaction between time (After MFP, Post, and 24-Post; 3 time points) and group. Abbreviations: SH, single hopping; VJH, vertical jump height; VJP, vertical jump power; DTKT, double-taped Kinesio taping; NTKT, normal-taped Kinesio taping; PG, placebo group; BMI, body mass index; MFP, muscle fatigue protocol.
